# On Improving The Computing Capacity of Dynamical Systems

**DOI:** 10.1038/s41598-020-65404-3

**Published:** 2020-06-08

**Authors:** Vasileios Athanasiou, Zoran Konkoli

**Affiliations:** Department of Microtechnology and Nanoscience, Chalmers University of Technology, Gothenburg, Sweden

**Keywords:** Computer science, Electrical and electronic engineering, Information theory and computation

## Abstract

Reservoir Computing has emerged as a practical approach for solving temporal pattern recognition problems. The procedure of preparing the system for pattern recognition is simple, provided that the dynamical system (reservoir) used for computation is complex enough. However, to achieve a sufficient reservoir complexity, one has to use many interacting elements. We propose a novel method to reduce the number of reservoir elements without reducing the computing capacity of the device. It is shown that if an auxiliary input channel can be engineered, the drive, advantageous correlations between the signal one wishes to analyse and the state of the reservoir can emerge, increasing the intelligence of the system. The method has been illustrated on the problem of electrocardiogram (ECG) signal classification. By using a reservoir with only one element, and an optimised drive, more than 93% of the signals have been correctly labelled.

## Introduction

**Figure 1:**
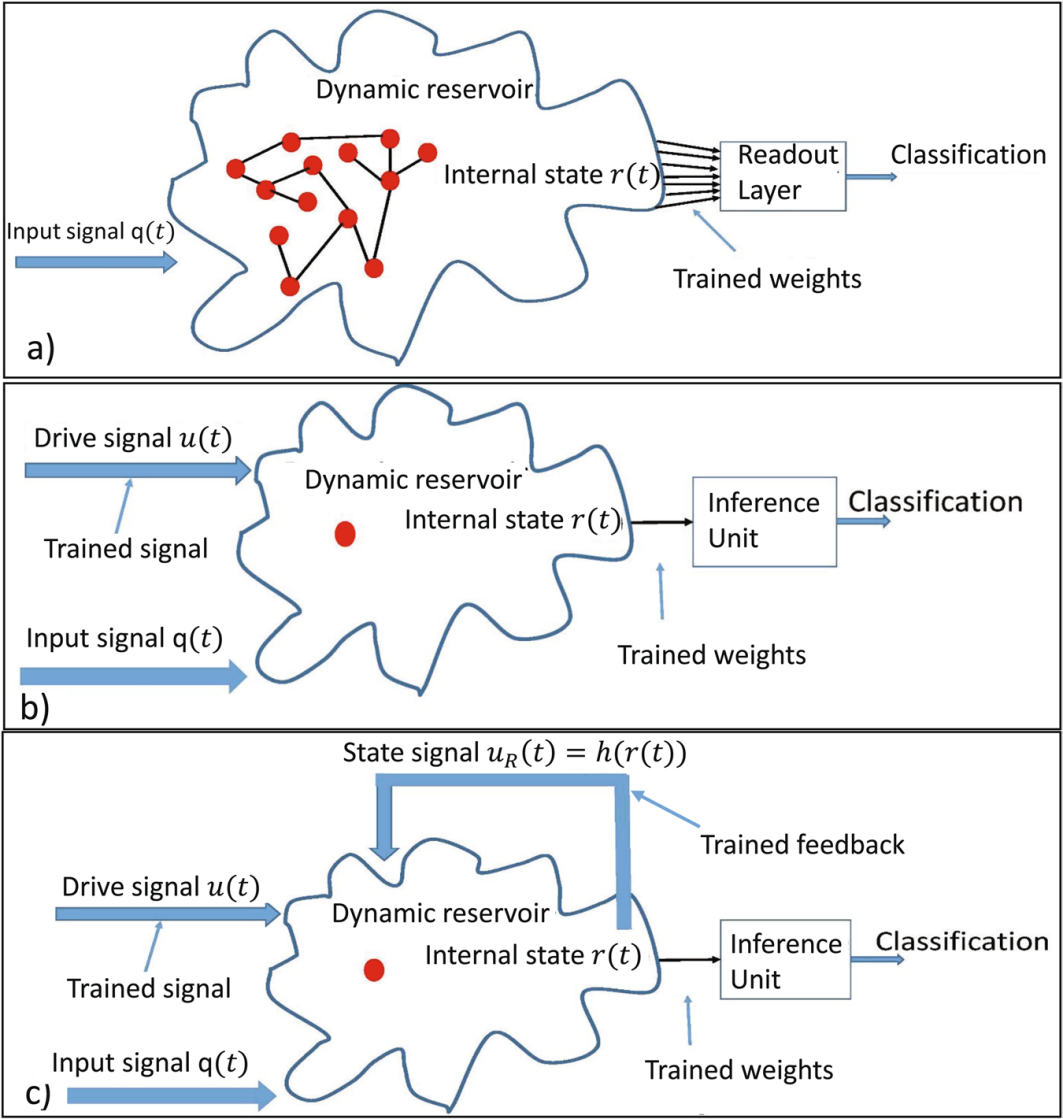
(**a**) The classical RC scheme. A dynamical system (reservoir) responds to the input *q*(*t*). The internal state of the reservoir *r*(*t*) is processed by a simple readout layer. The only part of the system being trained is the readout layer. (**b**) Same as for panel (a), but an auxiliary drive signal *u*(*t*) is provided that interacts with the reservoir. (**c**) Same as for panel (b) with an additional feedback mechanism *u*_*R*_(*t*) = *h*(*r*(*t*)) being included. The drive signal, the feedback mechanism and the weights are trained.

Reservoir Computing (RC) has been successfully used for solving plethora of temporal information processing problems, such as speech recognition or time series prediction and classification^1–3^. In Fig. 1a, we show the classical RC scheme in the context of signal classification. The internal state of the reservoir is defined as *r*(*t*) = (*R*_1_(*t*), *R*_2_(*t*), …, *R*_*N*_(*t*)) where *R*_*i*_(*t*) describes the state of an *i*-th element at a time instance *t*. For example, if the reservoir is built from neurons then *R*_*i*_(*t*) denotes the activation state of a neuron. Likewise, if the reservoir is a memristor network, then *R*_*i*_(*t*) represents a memristance value. Plethora of other elements have been suggested to build reservoirs (*cf*.^4^ and references therein). The set of all possible values of *r* constitutes the configuration space of the reservoir Ω.

The process of computation is realised by “feeding” an input signal *q*(*t*) to a dynamical system that represents the reservoir^5^. The external input *q*(*t*) “pushes” the state of the reservoir into a configuration *r*(*t*) according to a dynamic law $\dot{r}(t)=H(r(t),q(t))$, where the dot over a symbol denotes the time derivative, e.g. $\dot{r}\equiv dr/dt$. The configuration *r*(*t*) is the result of the computation performed by the reservoir computer. Note that the state of the system at a particular time instance *t* depends on the full history of the input signal up to that time point. Thus *r*(*t*) depends on all *q*(*t*′) with *t*′ < *t*. To emphasise this we write $r(t)= {\mathcal H} [q](t)$. Essentially, the reservoir works as a filter: it converts an uncountable series of values *q* into another series $r= {\mathcal H} [q]$. One can think of time as an index that can be used to access a particular value in such a series. In the following, when discussing specifically a one-element reservoir, to simplify the notation we write *r*(*t*) = *R*_1_(*t*) instead of *r*(*t*) = *R*_1_(*t*).

The state *r*(*t*) should also depend on the initial condition of the system *i*, and one ought to assume $r(t)= {\mathcal H} [q,\hat{r}](t)$. However, if the system exhibits the fading memory property then this dependence on the initial condition should be very weak: ${\mathcal H} [q,\hat{r}]\approx  {\mathcal H} [q]$. A few examples of dynamical systems that illustrate the fading memory concept can be found in literature^6^.

The reservoir is equipped with a readout layer *ψ* that assigns a label *y* to the reservoir state at every time instance $t:y(t)=\psi (r(t))$. The readout layer should be a simple structure, and it should not contribute substantially to the computation^7^; it is used to identify the region that the state has been driven to. For example, to realise a binary classification, one would use $y(t)=\psi ({r}_{w}(t))$ with ${r}_{w}(t)\equiv {\sum }_{i=1}^{N}\,{w}_{i}{R}_{i}(t)+{w}_{0}$, where *ψ* is a sigmoid-like function, and ${w}_{0},{w}_{1},\cdots ,{w}_{N}$ are free parameters.

The main claim is that under suitable reservoir conditions, one can realise *any* computation by using a *fixed* reservoir and by simply adjusting the readout layer achieve any desired functionality^8^. In the context of the binary classification example introduced above, this implies that any binary classification can be achieved by only adjusting the free parameters ${w}_{0},{w}_{1},\cdots ,{w}_{N}$. In principle, any dynamical system could be used like this provided that the system is complex enough.

It is intuitively clear that the computing power cannot come out of nothing. Indeed, in a genuine reservoir computing setup, the computing power does not reside in the readout layer, but it originates from the complexity of the reservoir^9^. In practice, to achieve this, one needs reservoirs with a large number of elements that feature recursive feedback mechanisms (intrinsic or externally engineered), both for hardware^10^ or software^3^ implementations. This intuitive understanding has been corroborated with rigorous mathematical arguments.

The most important formal criterion that makes the RC approach possible is that the dynamical system being used as the reservoir has to separate inputs^11,12^. The rigorous mathematical formulation is rather involved, and somewhat counterintuitive. In brief, assuming a fixed time reference *t*, a reservoir separates inputs if for any pair of distinct input signals *q* and *q*’ there is a readout layer ${\psi }_{q,q{\prime} }$ such that respective outputs $y(t)={\psi }_{q,q{\prime} }({r}_{q}[t])$ and $y{\prime} (t)={\psi }_{q,q{\prime} }({r}_{q{\prime} }[t])$ are different, where *r*_*q*_ = ${\mathscr{H}}$[*q*] and ${r}_{q{\prime} }={\mathscr{H}}[q{\prime} ]$. A pedagogical discussion on the mathematical background to the concept can be found in literature^13^. An intuitive formulation of the separability requirement is as follows. Assuming that the goal is to classify signals into a fixed number of *k* classes *c*_1_, *c*_2_, …, *c*_*k*_, the reservoir has to exhibit the following behaviour. If the input belongs to a class *c*_0_, then, the state of the reservoir *r* should be driven towards a region Ω_0_ of the configuration space; for an input of class *c*_1_ the state should be driven towards a region Ω_1_ and so on. The key requirement is that the regions Ω_0_, Ω_1_, …, Ω_*k*_ should not overlap and they ought to be compact so that the decision boundaries are simple^14–16^.

Input separation may not be possible if the reservoir were not complex enough. In machine learning, prior to the development of the deep learning paradigm, a specific practice for separating inputs has been considered. The practice features the use of additional input channels to achieve the desired separation, and is normally referred to as feature engineering^17–19^. In deep learning, feature engineering occurs spontaneously while training deep neural networks. Note that high quality features are found by training deep neural networks’ internal structure, which implies modification of the dynamical system used for computation. However, in RC, one is not allowed to adjust reservoir’s internal structure for training purposes. By assumption, one cannot modify the reservoir to increase the performance. This is particularly true in the context of the physical reservoir computing^7,13^ where, by assumption, one is interested in building computers from dynamical systems that cannot be easily modified. In this work, we suggest that it is possible to work around this problem.

Instead of modifying the dynamical system one can try to adjust the input that is being fed into the system. In particular, we demonstrate a generic method of building powerful reservoir computers by using an additional input feature, a drive signal. If providing an additional input feature contributes to input separation, then, one does not need to search for more complex dynamical systems with a larger number of interconnected reservoir elements. Instead, one can perform RC with the minimum amount of reservoir elements. The purpose of using an additional input feature is to achieve optimal input separation.

The key idea explored in this study is that a drive signal can be used to achieve advantageous correlations between input and reservoir’s state, increasing the performance of RC. If so, then a “recognizable” correlation between *r* and *q* is built in the sense that *r* can be used to infer about *q* without a substantial engineering overhead. If this can be achieved, then the drive and the reservoir can operate in synergy, so that the “intelligence” that should reside in the reservoir, is transferred into the drive instead, which could allow for reservoirs with smaller size^20^. In this context, we use the term “intelligence” as a measure of the computational resources needed to execute an algorithm, being often referred to as “logical depth”^21^. An implicit assumption is being made that a higher logical depth implies larger engineering overhead necessary to implement the algorithm on a computing machine. Therefore, the transfer of intelligence from the reservoir to the drive means that the need for reservoir’s computational resources can be reduced by using a drive signal.

The method works for dynamical systems that can be driven by an external signal. By assumption, the system responds to two signals, *q*(*t*) and *u*(*t*), where *q* is the signal one wishes to classify, and *u* features as an auxiliary additional input channel (feature). Using the notation introduced earlier, one can write $r(t)= {\mathcal H} [q,u](t)$. In Fig. 1, we show two implementations of the ideas discussed above in the context of a signal classification problem. **Implementation 1** (Fig. 1b): In this case, the reservoir is stimulated by a drive signal *u* and an input signal *q*. The dynamics of the reservoir is defined by $\dot{r}(t)=H(r(t),q(t),u(t))$. **Implementation 2** (Fig. 1c): The complexity of the system can be increased by adding a feedback, $\dot{r}(t)=H(r(t),q(t),u(t)+{u}_{R}(t))$, where the feedback signal *u*_*R*_(*t*) is a function of the reservoir state ${u}_{R}(t)=h(r(t))$. Note that *H* is considered fixed, and only once *H* is given, one chooses the appropriate implementation. This implies that those two implementations lead to two different (non-equivalent) pattern recognition algorithms, where implementation 1 is a special case of 2. For example, it is impossible to find a drive for implementation 1 that would mimic the behavior of implementation 2 if *h*(*r*(*t*)) ≠ 0. To do this, one would have to construct a drive that behaves as a filter, since the state of the reservoir *r* behaves in such a way. In particular, one would have to find a drive that has an intimate knowledge of the input signal *q*, but this is impossible as the input signal is not known *a priori*.

To find an optimal drive signal *u*, we suggest a generic training procedure that can be applied at any supervised learning problem or any reservoir. The system is optimised on a set of training data until the result of the computation agrees with the desired functionality. The ability of the system to generalise is examined by using a separate set of test data.

The training procedure consists of two phases. In the first phase, the drive signal and the feedback (if used) are optimised to achieve maximum input separation, without considering the readout layer. If the reservoir separates inputs, then, a simple readout layer (*e.g*. linear classifier) could be trained to successfully classify inputs by reading the reservoir’s state^11^. In the second phase, only the readout layer is optimised, by keeping the drive signal and the feedback function found from the first phase. The advantage of considering the two phases of training is that the readout layer can be optimised offline. The states of the reservoir are generated under the optimum drive and feedback (if used). Then, the readout layer is trained with supervised learning to infer the correct class by reading the reservoir states.

**Phase 1:** To measure input separation we use the separability index which is introduced and described in detail in the methods section. For a single-element network and a binary classification problem with classes of input signals *c*_0_ and *c*_1_ the separability index is defined as

1 $$\nu [u]={\langle d[u,{q}_{0},{q}_{1}]\rangle }_{{q}_{0}\in {c}_{0},{q}_{1}\in {c}_{1}}$$

where ${\langle \mathrm{..}.\rangle }_{{q}_{0}\in {c}_{0},{q}_{1}\in {c}_{1}}$ denotes the geometric mean over different input signals *q*_0_ and *q*_1_ that characterise each input class *c*_0_ and *c*_1_ respectively. The variable *d*[*u*, *q*_0_, *q*_1_] denotes a typical distance between trajectories that are generated when the system is exposed to each pair of input signals *q*_0_ and *q*_1_ under a drive *u*.

Trajectories are analysed over an extended time interval [0, *T*] where *T* denotes the observation time. A typical distance is computed as

2 $$d[u,{q}_{0},{q}_{1}]=\bar{R}[u,{q}_{0}]-\bar{R}[u,{q}_{1}]|$$

with

3 $$\bar{R}[u,q]=\frac{1}{T}{\int }_{0}^{T}\, {\mathcal H} [u,q](t)dt$$

note that the average over time is computed first, followed by the computation of the difference between the average memristance values. In methods, it is explained why it would be wrong to perform these operations in the reverse order.

The degree of trajectory separability can be controlled by the drive signal *u*. Thus, in the first training phase, the goal is to find the optimal drive signal u_*_ = maxarg_*u*_*v*[*u*] and the feedback function h_*_ (if used) that achieves the maximal trajectory separability. We use a genetic algorithm optimisation, which is described with details in methods, with the separability index *v*[*u*] as the fitness function.

**Phase 2:** The readout layer implementation used differs somewhat from the central RC dogma. The purpose of the first phase of training is to maximise the separation of inputs over the whole time interval *T*, which features the computation of average memristance values ${\bar{R}}_{i}={T}^{-1}{\int }_{0}^{T}\,{R}_{i}(t)dt$. Therefore, instead of the classical readout layer, the inference unit performs the assessment at the time instance *T*: $y(T)=\psi ({\bar{r}}_{w})$ with ${\bar{r}}_{w}\equiv {\sum }_{i=1}^{N}\,{w}_{i}{\bar{R}}_{i}+{w}_{0}$. When compared to the classical RC readout layer, the computational cost of implementing such a unit is marginal. The idea to construct the readout layer by using such averages have been verified independently in separate studies^22^.

For technical reasons we assume $\psi ({\bar{r}}_{w})={\bar{r}}_{w}$. This allows the use of the least square method. In particular, for a binary classification problem, the output should be close to +1 (−1) when a signal from class *c*_0_ (*c*_1_) is applied, and we simply minimise the prediction error by varying *w*_0_, *w*_1_, …, *w*_*N*_. The decision by the inference unit is considered as the simplest possible: if *y*(*T*) > 0 then infer class *c*_0_ otherwise infer class *c*_1_.

The dynamical system used consists of a single memristor and an optional feedback unit. A memristor is a non-linear, passive, two-terminal component with a time-varying resistance often being referred to as the memristance *R*(*t*). The memristor element is suitable for temporal information processing since it exhibits the filter property: The memristance value at a specific time instance depends on the whole history of the applied voltage signal up to that time. Few applications of memristor networks for temporal information processing purposes can be found^2,23^.

To illustrate the method, a simple Pershin Di Ventra model^24^ is used. The memristance *R*(*t*) changes depending on the voltage signal Δ*V* that is applied across the element according to a simple law. If −*V*_*thr*_ < Δ*V* < *V*_*thr*_, then the memristance changes as $\dot{R}=\alpha \Delta V$. For Δ*V* < −*V*_*thr*_ or Δ*V* > *V*_*thr*_, $\dot{R}=\beta \Delta V+const$; *α*, *β* are device dependent parameters, *const* is a constant value and usually *α* ≪ *β*. The memristance is bounded between the lowest value *R*_*min*_ > 0 and the maximum value *R*_*max*_. This can be written as:

4 $$\dot{R}(t)=f(\Delta \,V(t),\beta )\Theta (R(t),\Delta \,V(t))$$

with

5 $$f(\Delta \,V,\beta )=\beta \Delta \,V+\frac{1}{2}\,(\alpha -\beta )\cdot (|\Delta \,V+{V}_{thr}|-|\Delta \,V-{V}_{thr}|)$$

and

$$\Theta (R,\Delta \,V)=\{\begin{array}{ll}0, & {\rm{if}}\,\Delta \,V=0\\ \theta ({R}_{\max } > R), & {\rm{if}}\,\Delta \,V > 0\\ \theta (R > {R}_{\min }), & {\rm{if}}\,\Delta \,V < 0\end{array}$$

where *θ*(*R*_*max*_ > *R*) is zero unless the condition in the argument is satisfied, and likewise for *θ*(*R* > *R*_*min*_).

**Figure 2:**
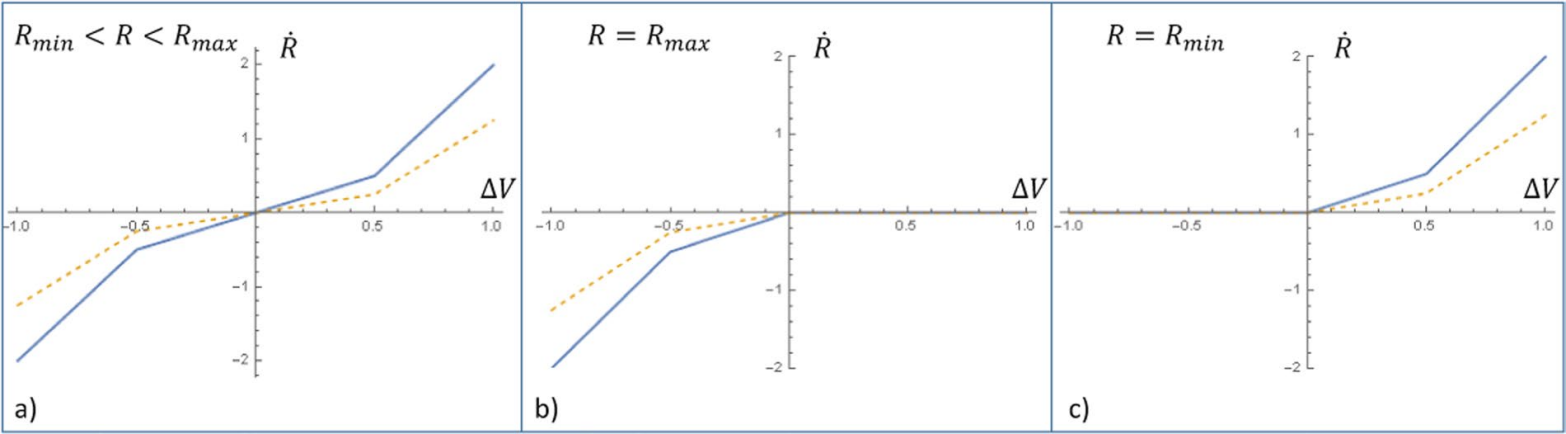
The memristance rate of change $\dot{R}$ is plotted against the volage difference Δ*V* for values of parameters *β* = 3.0, *α* = 1.0, *V*_*thr*_ = 0.5 (thick line) and *β* = 2.0, *α* = 0.5, *V*_*thr*_ = 0.5 (dashed line). The plot is given for three different cases of the value *R*(*t*) when (**a**) *R*_*min*_ < *R* < *R*_*max*_, (**b**) *R* = *R*_*max*_ and (**c**) *R* = *R*_*min*_.

**Figure 3:**
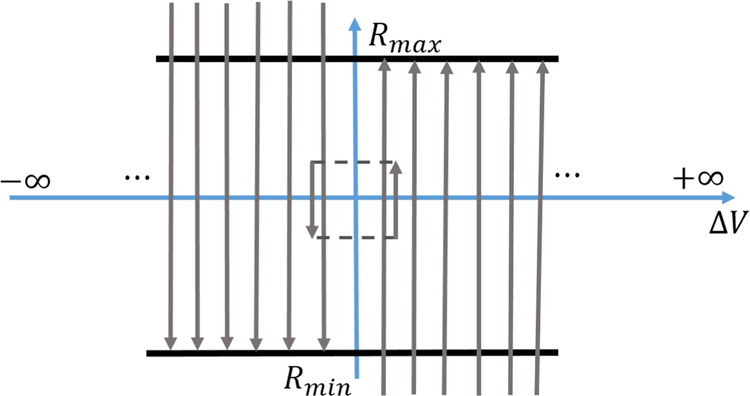
The flowlines of the dynamical system. The small rectangle shows the path the system would take under a square wave voltage input.

Figure 2 shows how $\dot{R}$ depends on *R* and Δ*V*. The dependence of *f* on the applied voltage Δ*V* (*R*_*min*_ < *R* < *R*_*max*_) is shown in panel a). The flowlines of the ordinary differential equation system are illustrated in Fig. 3. When Δ*V* > 0, then the memristance increases until *R* = *R*_*max*_ is reached. When Δ*V* < 0, the memristance decreases until *R* = *R*_*min*_.

In this study, the parameters *α*, *V*_*thr*_, *R*_*min*_ and *R*_*max*_ are always kept fixed. More realistic memristor models are available. For example, real devices can exhibit asymmetry in the memristance change ($\dot{R}(\Delta \,V)\ne \dot{R}(\,-\,\Delta \,V)$) and non-linear rate of memristance changes^25^. However, the model used here is known to reproduce most of the experimental results.

The drive signal *u*(*t*) is expressed as a Fourier series with nine amplitudes and a base frequency (details provided in the methods section). The feedback function is defined as *u*_*R*_(*t*) = *h*(*R*(*t*)) = *p*_1_*R*(*t*) + *p*_0_. The ten parameters of the drive signal and the parameters *p*_1_, *p*_0_ are optimised in the first phase of the training procedure.

Two options of how the input signal *q*(*t*) interacts with the reservoir are considered. **Option 1:** It is natural to assume that the input signal influences the *β* parameter of the memristor model making it time dependent: *β*_*q*_(*t*) = *m*_1_*q*(*t*) + *m*_0_. Such a model has been explored before^23^. **Option 2:** Another option is to assume that the input signal acts as an external voltage source^2^ *u*_*q*_(*t*) with *u*_*q*_(*t*) = *k*_1_*q*(*t*) + *k*_0_ and *β* kept fixed at the value *β*_*c*_. These two options (Options 1 and 2) along with the two implementations (Implementations 1 and 2) result in the four models I1O1, I2O1, I1O2 and I2O2. The dynamical equations for those models are summarised in Table 1.

**Table 1:** The equations which describe the dynamics of the models I0O1, I1O1, I2O1, I0O2, I1O2 and I2O2.

	Dynamics
I0O1	$\dot{R}(t)=f({u}_{c}(t),{\beta }_{q}(t))\Theta (R(t),{u}_{c}(t))$
I1O1	$\dot{R}(t)=f(u(t),{\beta }_{q}(t))\Theta (R(t),u(t))$
I2O1	$\dot{R}(t)=f(u(t)+{u}_{R}(t),{\beta }_{q}(t))\Theta (R(t),u(t)+{u}_{R}(t))$
I0O2	$\dot{R}(t)=f({u}_{q}(t),{\beta }_{c})\Theta (R(t),{u}_{q}(t))$
I1O2	$\dot{R}(t)=f(u(t)+{u}_{q}(t),{\beta }_{c})\Theta (R(t),u(t)+{u}_{q}(t))$
I2O2	$\dot{R}(t)=f(u(t)+{u}_{q}(t)+{u}_{R}(t),{\beta }_{c})\Theta (R(t),u(t)+{u}_{q}(t)+{u}_{R}(t))$

We compare the performance of these four models with a set of simpler models in which neither a drive signal nor a feedback function are optimised. In such a way one can test the performance of a reservoir when no additional input features are provided. This reveals a “raw” intelligence of a reservoir. Then, the increase in the additional computational power provided by an additional input feature can be analyzed. The dynamical equations of the models I0O1 and I0O2 are given in Table 1. The model I0O1 results from option 1 when neither a drive signal nor a feedback function is optimised. In this case, the reservoir is always driven by the same drive signal *u*_*c*_ and there is no feedback *u*_*R*_(*t*) = 0. The model I0O2 results from option 2 without providing any drive signal nor a feedback: *u*(*t*) = 0 and *u*_*R*_(*t*) = 0.

## Results

As a source of data, the labelled data set of ECG signals “ECG5000” from the UEA and UCR Time Series Classification Repository^26^ is used. Usually, in the literature, ECG signals are classified into many classes^27^. However, since the reservoir consists of only one memristor element, a simple problem of binary signal classification is investigated. Including more classes would require larger memristor networks. Thus only two classes are considered, class *c*_0_ (healthy heart) and class *c*_1_ (heart problems). The strategy of using the single memristor for ECG signal classification is simple to describe: the memristance ought to be driven towards *R*_*min*_ or *R*_*max*_ depending on whether the input signal belongs to *c*_0_ or *c*_1_ respectively. If this can be achieved, classification can be performed by simply checking whether the memristance value exceeds a pre-defined threshold. The same type of thinking applies to more complex classification problems with many classes. For example, with a two-memristor network with *r* = (*R*_1_, *R*_2_) a four-class problem could be solved. The state could be driven towards four different regions (*R*_*min*_, *R*_*min*_), (*R*_*min*_, *R*_*max*_), (*R*_*max*_, *R*_*min*_), and (*R*_*max*_, *R*_*max*_).

In the training procedure, 40 signals with the label *c*_0_, and 40 with the label *c*_1_ have been used. The training procedure for each model (I1O1, I2O1, I1O2 and I2O2) resulted in an optimum drive signal: *u*_11_ for I1O1, *u*_21_ for I2O1, *u*_12_ for I1O2 and *u*_22_ for I2O2. The two optimal feedback functions were found too: *h*_21_ for I2O1 and *h*_22_ for I2O2.

The ability of the trained models to generalise has been validated on a separate set of test data: 740 signals with the label *c*_0_, and 740 signals with the label *c*_1_. The trained models were exposed to those signals, and the percentage of correct label assignments was counted. The quality of recognition is described in terms of the success rate 0 ≤ *S* ≤ 1 being defined by the percentage of correct signal classifications. Thus *S* ≈ 1 indicates a good classifier.

In this work, the set of test data has been considered to be much larger than the set of training data (740 ≫ 40). The reason is to test the performance of the proposed models in the worst case when few training examples are available. It has been shown that RC performs relatively well with a few training examples because RC can extract general features from the training data set^28,29^. Choosing data sets and their size can be crucial in machine learning for improving accuracy of models. One could try different sizes of data sets and the performance of models would also depend on the choice of data sets^30^. Investigating the right choice of data sets and their size falls out of the scope of this article.

An ECG signal has a natural time reference, the top of the QRS peak (regardless of the class). Thus in the database all signals are aligned so that their QRS peaks coincide. To investigate whether the models can be used for classifying non-synchronised ECG signals, we intentionally modified the phase of each signal in the training and the test data sets by random shifts in time. Thus we distinguish two major groups of data sets: *aligned* versus *asynchronous*.

In the simulations, since ECG signals are quasi-periodic, the observation time was taken to be ten periods, *T* = 10 periods. The reservoir has the chance to “absorb” in its state extended information about the input over a longer time interval. In such a way, small features of the input signal that might be ignored by visual inspection, can accumulate over time and be detected. Observing the signal over several periods gives the reservoir necessary time to distinguish important trends from insignificant fluctuations.

Since the memristance value is bounded, it is useful to normalise the separability index *v* in a range of possible values between 0 and 1. When *v* = 1 this signals a maximum input separation. Any two input signals from the two different classes drive the state (on average) to its opposite extreme bounds *R*_*min*_ or *R*_*max*_. The value *v* = 0 indicates that there is no correlation whatsoever between the states’ values and the input signal *q*. For example, it is even possible that the state is driven on average to the same region of values for both classes of inputs.

### Frozen input layer

The device parameters *m*_1_, *m*_0_, *k*_1_, and *k*_0_ used in simulations have been kept fixed. The values of *m*_1_ and *m*_0_ were chosen so that *β*(*t*) has a mean value close to *β*_*c*_ and is always positive. The values of *k*_1_ and *k*_0_ were chosen so that the voltage signal *u*_*q*_(*t*) is bounded in the [−10, 10] interval. The drive signal *u*_*c*_ was chosen as a square wave pulse with +1 the first half and −1 the second half of each period. The values for all parameters used in the simulations are provided in the methods section.

**Figure 4:**
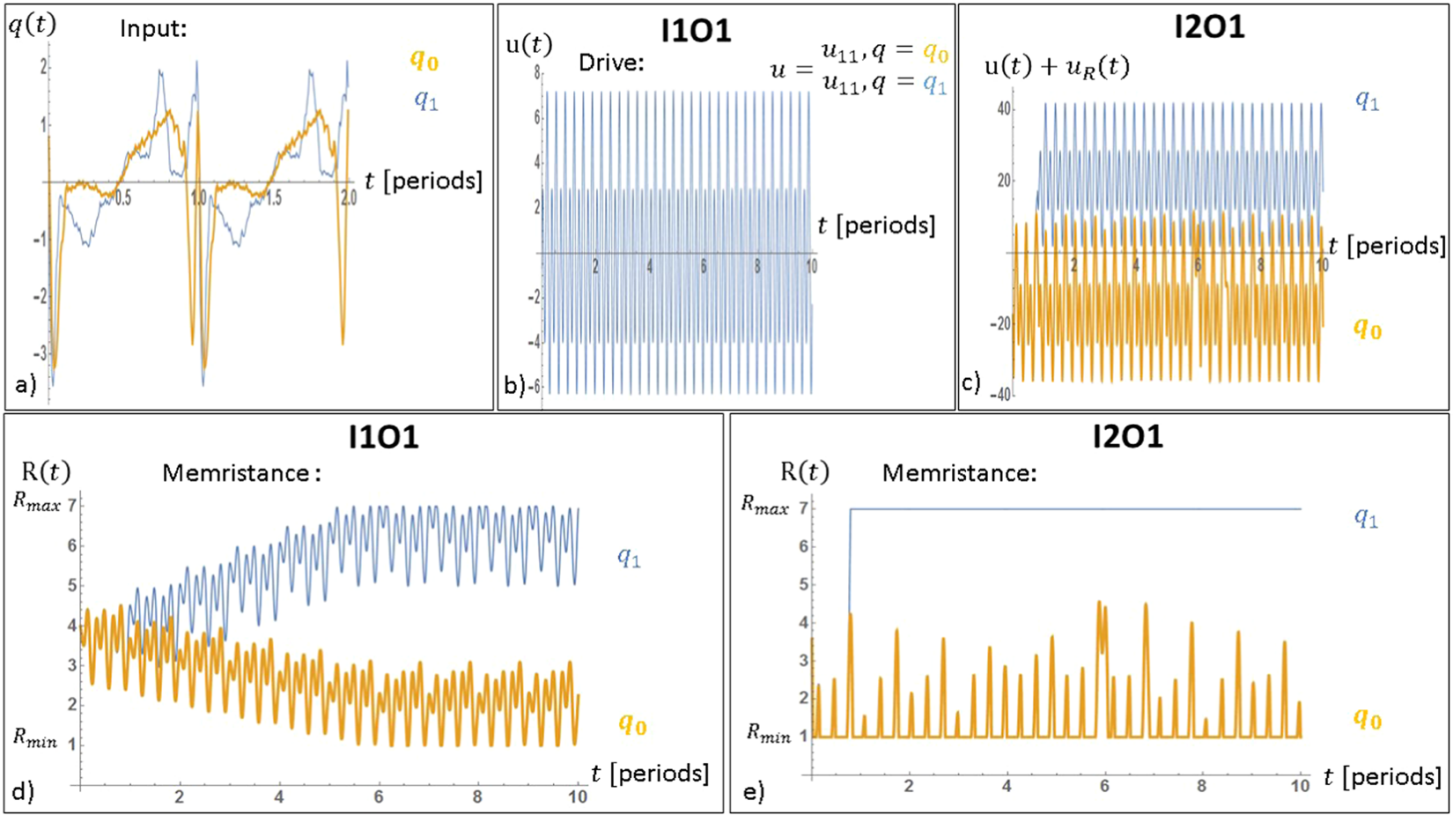
(**a**) An example of two input signals taken from for the two classes of interest. Panels (b,c) depict the optimised drive signals *u*_11_ and *u*_21_. Panel (d) shows the memristance behavior of model I1O1 when exposed to *u*_11_ and the two input signals shown in (**a**). (**e**) The memristance as function of time for model I2O1 when exposed to *u*_21_, with the feedback function *h*_21_ and the two input signals shown in (**a**).

In Fig. 4, the phase space separation is shown as it was achieved with the optimum models I1O1 and I2O1 under two input signals from the different classes. Those input signals are shown in panel a) covering two periods in time, to be referred to as *q*_0_(*t*) and *q*_1_(*t*) for class *c*_0_ and *c*_1_ respectively. In b), only one curve is shown, the optimised drive signal *u*_11_(*t*). This drive signal governs the applied voltage signal across the memristor and is independent of the input signal. In d), the memristance trajectories *r*_0_(*t*) = *H*[*r*_0_, *q*_0_, *u*_11_](*t*) and *r*_1_(*t*) = *H*[*r*_1_, *q*_1_, *u*_11_](*t*) are shown for the model I1O1.

The phase space separation is evident: the trajectories *r*_0_(*t*) and *r*_1_(*t*) are driven towards two different regions of the state space: *r*_1_(*t*) ≈ *R*_*max*_ and *r*_0_(*t*) ≈ *R*_*min*_ for *t* → ∞. It is important to notice that the memristor model is exposed to the same drive *u*_11_ for both inputs *q*_0_ and *q*_1_; the machine can never “know” which input signal it will have to analyse. Yet, the drive posses an intrinsic “intelligence” that works in synergy with the memristor element, to achieve the phase space separation. The drive signal *u*_11_ has been found so that when *q* = *q*_0_ (*q* = *q*_1_) the memristance is driven to a region close to *R*_*min*_ (*R*_*max*_) for *t* → ∞. Note here that *u*_11_ (panel (b)) has both positive and negative values. If *u*_11_ was only positive (negative) then *R*(*t*) ≈ *R*_*max*_ (*R*(*t*) ≈ *R*_*min*_) and *R*(*t*) would not be separable. Therefore, *u*_11_ has been found with the right amount and synchronisation of positive and negative values to achieve input separation.

In panel c), the voltage signal across the memristor model is shown when the model I2O1 was simulated with *u*_21_(*t*), *h*_21_ and the input signals shown in a). Because of the feedback, the voltage across the memristor *u*_21_(*t*) + *u*_*R*_(*t*) is different for the two different applied inputs. Again, the phase space separation is evident, as shown in panel e). In fact, one can pick any pair of signals from classes *c*_0_ and *c*_1_ and, provided the same optimised *u*_11_, *u*_21_, and *h*_21_ are used, the separation property will likely hold. Thus one can think of *u*_11_, *u*_21_, and *h*_21_ as the device configuration “parameters”.

The fact that input separation is evident in panels d) and e) shows why the two training phases suggested in this work operate in synergy. The task of genetic algorithm optimisation was to find a drive signal so that phase space separation occurs for most of the signals in the training data set. If so, then, one can simply build a linear classifier for inferring the class of the input signal. For example, by observing those two panels, one could build a linear classifier with a bare eye.

Additionally, it is of interest to notice that the response of the device was faster for the model I2O1 than the model I1O1. For example, in e) one can see that phase space separation occurred already at the first period of the input signal. However, in d) more periods were needed for the memristance to be driven towards its bounds. This is owed to the usage of a feedback function. An additional usage of a feedback function resulted in faster responses.

The values of the separability index *v* and the success rates of the optimised circuits are summarised in Table 2. The left panel summarises the results of the analysis for synchronised data set signals. The right panel is for asynchronous signals.

**Table 2:** Optimised *v* values obtained during the training process, and success rates *S* during the test phase.

	*v*	*S*		*v*	*S*
I0O1	0.126	87.2%	I0O1	0.093	47.8%
I1O1	0.501	97.9%	I1O1	0.204	55.4%
I2O1	0.839	93.2%	I2O1	0.729	87.9%
I0O2	0.002	51.9%	I0O2	0.004	75.3%
I1O2	0.059	97.8%	I1O2	0.027	45.0%
I2O2	0.890	96.1%	I2O2	0.441	79.0%

Left panel: all signals are aligned (with the QRS wave); right panel: non-aligned signals.

#### Aligned signals

For the aligned signals in Table 2, a larger separability index *v* was obtained for the models with a feedback mechanism (I2O1 and I2O2) than the ones without feedback (I1O1 and I1O2). The lowest separability indexes were obtained for the models I0O1 and I0O2 where neither a drive nor a feedback were optimised. To judge whether a larger separability index means a better training process, one should test the optimised models with a test data set. In the left panel, all the success rates with optimised input features (I1O1, I2O1, I1O2 and I2O2) were found *S* > 93.2% indicating that these or similar models can be used for classifying ECG signals.

It is important to notice here that the success rates were found smaller for the models with optimised feedback than the models with just optimised drive signal. This happened due to overfitting, a problem which usually occurs in machine learning: The extra parameter to be optimised (feedback) was so powerful that the models fit too much on details of the training data set that were not generally features of other input signals of the test data set.

The success rates for models with just a drive signal (I1O1 and I1O2) were found larger than the models without any drive signal (I0O1 and I002). In particular, by training a drive signal, the success rates improved from 87.2% to 97.9% and from 51.9% to 97.8%. Therefore, training a drive signal resulted to exceptional success rates which could not be achieved by just using a memristor element.

Additionally, for the model I1O2, even though the training process resulted in a very low *v* = 0.059, the success rate was large (97.8%). This happened because it was still possible to construct a readout layer which classifies most of the input signals correctly. By inspecting closer the state *R*(*t*) over time (not shown), we saw that although the instantaneous state *R*(*t*) was driven to overlapping regions, the average value of the state was separable. This is one of the advantages of using a readout layer which averages the state over time. For example, if the memristance values were driven on average to the numerical values 3.10 and 3.00 for inputs of class *c*_0_ and *c*_1_ respectively, then the separability index would be small but it would still be possible to construct a readout layer: if the average memristance is larger than 3.05 then infer class *c*_0_ otherwise infer class *c*_1_.

These results indicate that a single memristor model equipped with an optimised voltage driving signal can be used for ECG signal classification. Note that the drive needs to be trained only once during the first phase of training, on a known set of signals, but the device can be used on unknown signals. However, we reiterate that the downloaded data set consists of signals with the same phase, (see Fig. 4a)). In practice, for online classification, one would need an online QRS detector^31^. QRS detectors are used to recognise the phase of ECG signals by identifying the part of the ECG signal which changes fastest in time. Moreover, the task of signal classification would be harder if there was no information regarding the phase of the signals, for example for embedded computation where every piece of extra equipment is a problem, one might want to remove a QRS detector unit. Can the system still classify with such a high success rate if there is no fixed time reference?

#### Asynchronous signals

The separability indexes *v* and the success rates in the right panel in Table 2 for non-aligned signals were found smaller than their aligned counterparts listed in the left panel. This is expected because now the models should be smarter and infer correctly independently of the input signal phase. The models without a feedback mechanism (I1O1 and I1O2) performed worse with success rates 55.4% and 45.0% respectively. Including and optimising a feedback mechanism (models I2O1 and I2O2) improved both the training procedure (index *v*) and the testing one (success rate *S*). The separability indexes from I1O1 to I2O1 and I1O2 to I2O2 improved from 0.204 to 0.729 and from 0.027 to 0.441 respectively. Additionally the success rates improved from 55.4% to 87.9% and from 45.0% to 79.0%. Therefore, here, contrary to the results of using the initial data set (in the left panel in Table 2) there was no over-fitting: The extra parameter to be optimised (feedback) was so powerful that the models fit better on some features of the training data set which are also features of the test data set.

### Optimised input layer

The classification performance can be improved if there is an option to adjust the input layer parameters *m*_1_, *m*_0_, *k*_1_, or *k*_0_. While this is clearly possible for *in silico* implementations, it might be possible for hardware implementations too. The values of the separability index *v* and the success rate when the input layers were additionally optimised are shown in Table 3 for both aligned (left panel) and non-aligned signals (right panel).

**Table 3:** Optimised *v* values and success rates *S* for the system with the optimised input layer.

	*v*	*S*		*v*	*S*
I0O1	0.239	82.0%	I0O1	0.205	48.2%
I1O1	0.818	98.6%	I1O1	0.290	55.5%
I2O1	0.774	90.1%	I2O1	0.640	91.0%
I0O2	0.042	88.6%	I0O2	0.046	42.9%
I1O2	0.211	90.8%	I1O2	0.186	90.3%
I2O2	0.843	96.3%	I2O2	0.783	93.1%

Left panel: all signals are aligned (with the QRS wave); right panel: non-aligned signals.

By comparing the results in Table 2 to the ones in Table 3, the following key results were found regarding the additional optimisation of a simple input layer:

- The largest success rates across all models is *S* = 98.6% for the aligned signal data set (Table 3, the second row), and *S* = 93.1% for the non-aligned signal data set (Table 3, the sixth row).
- Larger success rates were achieved for the non-aligned signals when the input layer was optimised (*S* > 90% in Table 3 versus *S* < 90% in Table 2).
- In contrast to the above, for the aligned data set, the input layer optimisation does not improve the success rate uniformly across all models (*cf*. the S values in the left panels of Tables 2 and 3). This could be an instance of overfitting: the input layer optimisation works well on the training data but does not generalise (towards the test data).
- A single memristor model without optimising a drive signal (models I0O1 and I0O2) cannot be used to classify the ECG signals since *S* < 90% for both frozen and optimised input layers. The only way to achieve *S* > 90% was to provide an additional input feature such as a drive signal or a drive signal with a feedback function. For example, in Table 3 right panel, models I1O2 and I2O2 performed better than I0O2 and model I2O1 performed better than I0O1.

An interesting finding concerns the question whether the addition of a feedback function improves *v* and *S*. It depends on how hard is the problem. For easier classification problem, when the signals are aligned, the feedback does not always improve the accuracy of the prediction. However, for a harder problem, when the signals are not aligned, the addition of the feedback always improves both *v* and *S*. For example, I2O1 did not perform better than I1O1 when the signals were aligned (see the left panel in Table 3). Even though I2O1 is an extension of I1O1, neither a larger index *v* nor a larger *S* was found for I2O1. This has probably happened because of convergence problems during the optimisation. It was hard to optimise both the input layer and the drive signal. Probably, better optimisation algorithms should be tested in future, such as optimising one layer at a time, *e.g*. optimising the input layer and keeping fixed the feedback and the drive signal *etc*.

## Discussion

A novel classification method has been suggested that can be used to increase the intelligence of pattern recognition devices. The method requires modest resources to implement. The approach has been illustrated in the reservoir computing context, but the method could be easily used in other pattern recognition setups.

A typical pattern recognition device is a dynamical system that accepts an input signal and informs about which class the signal belongs to by producing a label, being the output of the computation. The key idea is to equip a machine learning system (the reservoir) with an auxiliary input channel, the drive signal, that is external to the system and easy to control. This signal can be optimised so that the information about the signal one wishes to analyse (the input signal) can be efficiently embedded into the reservoir state. Then, the reservoir state can be analysed to infer the information about the input.

An intuitive way of understanding the concept is to envision a theatre performance. A prompter in a theatre helps an actor that forgot a line. The prompter is familiar with the intrinsics of the play, and has an overall understanding of the right timing of the narrative. In a similar way, the optimised drive provides clues that accumulate over time and increase the overall intelligence of the system.

The method can be exploited in two ways. First, in the bottom-up approaches, when the reservoir is engineered from scratch, the intelligence that normally resides in the reservoir can be moved to the drive. This would allow engineering reservoirs with smaller size, without reducing the computing capacity of the device. Second, the intelligence of an existing system could be increased, especially for reservoirs that built in a top-down manner, that are not engineered from scratch or once engineered are not meant to be modified. The respective examples might include an amorphous structure (e.g. to be used for *in materio* computation), or an echo state network (essentially a random neural network with feedback links).

Our approach offers a series of practical advantages. The reported successes rates have been obtained with rather modest resources: the machine learning system consisted only of a simple memristor element, and an optimised drive signal. Thus, it has been demonstrated that extra intelligence can indeed be provided through the external drive since a single memristor (models I0O1 and I0O2) cannot be used to solve such a classification problem. The results of our work point to the exciting possibility that there is a potential synergy between the reservoir state dynamics and the external input signal profile (the signal one wishes to analyze) in terms of the information they could potentially share. This synergy can be exploited, but it is deeply hidden, scattered over time, as small clues that a traditional pattern recognition device ignores. The drive signal can be used to unlock that hidden potential. Clearly, such a synergy might not exist for every information processing problem (a particular reservoir-input combination), but it seems that for the problem that has been investigated it is there: The presence of the drive had much stronger effect on the aligned signals with a fixed time reference. Further, it is true that the presence of the drive imposes some limitations on the system design. However, arranging for an extra input channel ought to be possible in general.

To demonstrate the method, we used it to classify ECG signals. The method classifies the ECG signal with success rate 98.6% (93.1%) for aligned (non-aligned) signals. The reported success rates fall roughly in the range of other state of the art methods. For example, it has been reported that for the data set “ECG5000”, the best performing algorithm “COTE”, achieved a success rate of 94.61%^32^. Other state-of-the-art approaches, *e.g*. deep learning networks^33^, have reported similar success rates. Interestingly, the state of the art success rate has been achieved despite the fact that rather modest resources were used to implement the classification algorithm. Naturally, such a comparison is not entirely fair since we use the lowest possible number of classes (two). In contrast, the COTE algorithm was applied on the set of aligned signals with five-classes. Likewise, Hannun *et al*.^33^ have solved a ECG classification problem with twelve classes and different ECG data sets. Thus it is likely that the success rates reported in here would deteriorate if one would increase the number of classes. However, to mitigate this one can consider using more complex memristor networks. Still, the fact that the success rates fall in the same range illustrate the potential of the method.

Since we considered just one memristor element it did not make much sense to work classification problems with more than two classes. The method can be easily applied to any multi-class pattern recognition problem, however. To do this, as already mentioned, one could consider using more complex dynamical systems. But there are other practices used in machine learning that might be used with an advantage. One could also consider a combination of several classification systems similar to ones suggested in this work. For example by, again, using a single memristor equipped with one linear readout layer. In particular, one system could be trained for a different task, *e.g*. to classify if a signal belongs to class 1 or 2, another system if a signal belongs in class 1 or 3, *etc*. Another option would be to use current work as models for trying ensemble learning methods^34^. All those ideas are left for future implementations.

The training data set (80 signals) was much smaller in size than the testing data set (1480 signals), and still the system performed very well. The findings of our method agree with other findings^28,29^ that RC works well with a few number of training examples. Generic features can be extracted from the training data set since a few parameters are trained because reservoir’s internal structure is not trained. Similarly, in our work, the fact that the drive was parameterised with very few parameters (and a limited training data set) suggests that the hidden correlations we seek to explore are in some sense global, and do not require detailed drive tuning to access. It is possible that a sub-optimised drive could do the job as well as the fully optimised one. In fact, this has also been corroborated by inspecting the genetic algorithm optimization steps. There is no reason to believe that such a global synergy could not be found and exploited in other systems.

The biggest challenge with the method is the process of training the drive. For very large systems with many elements the drive optimization procedure might be a problem. One would have to compute distances between points in a space with a very large dimension over many time instances. However, this issue could be mitigated in several ways.

First, to train the drive, there is no need for a rigorous supervised learning approach during this phase. Let us illustrate this on a thought experiment. Assume that the goal is to apply the approach for the pattern recognition problem where the goal is to use an artificial neural network. It is true that during the drive optimization step the user needs to have an access to labelled data, but not in the same way as for supervised learning approaches. To train an artificial neural networks one uses labels actively to train “inside” of the network, i.e. adjust its weights. Our use of the labels is more passive, we operate from the “outside”. The main advantage of the suggested approach is that an easily adjustable external signal needs to be manipulated instead of the internal network weights. This might not matter for *in silico* implementations but it might be a decisive difference for hardware implementations, especially if one cannot engineer the reservoir easily.

Further, the fact that a sub-optimal drive might work opens for several options for building scalable separability optimization techniques that can be used on large systems. An obvious option is to reduce the training data set size: The size of the training data set influences the number of distance computations, and as argued above, the training data set with limited size was sufficient to optimise the drive. Another option to explore is to estimate separability in some other way, without computing distances between all trajectories (and trajectory points), when forced working with large data sets. Then, one might try to use a sampling technique in which not every distance has to be computed. For example, instead of computing the distance among the full set of trajectory points, one picks few sampling points at random, *e.g*. when the average trajectory position is used to estimate distances. Such an average could be efficiently estimated efficiently by random sampling.

It has been shown that including more reservoir resources (in addition to the external drive signal) such as feedback mechanisms or input layers significantly improved the performance of the device. These options are recommended for software applications since it would only cost the training of just few additional free parameters. However, for hardware implementations, those options should be considered wisely: these additional resources imply additional engineering overhead, *e.g*. in terms of energy consumption.

While the method is demonstrated theoretically on a simple memristor model, it is likely that such recognizable correlations can be exploited in other dynamical systems. The extensions towards other systems ought to be straight forward, at least in principle. It is possible that a novel pattern recognition paradigm might emerge at the interface between the theory of complex dynamical systems, and state-of-the-art machine learning methods, by further exploring (developing and applying) the method suggested in here.

## Methods

To perform the first phase of the training procedure, we introduce a separability index and a genetic algorithm optimisation. The separability index acts as the fitness function of the optimisation. The details for calculating the separability index are presented in the first subsection. The optimisation algorithm is explained in the second subsection. Details of implementing numerical simulations are presented in the third subsection.

### Separability index

The separability index *v* is introduced as a tool for quantifying the ability of a reservoir to separate inputs. Several such measures have been considered in the literature^14–16^. The measure suggested in this study is specifically tailored towards measuring the degree of collaboration between the elements.

One assumes a drive signal *u* and generates all possible trajectories for a given training data set which is a set of input signals with a corresponding label each one. The separability index *v* is computed by estimating typical distances between the trajectories. If typical distances are large then *v* should be large too. This implies that *v* depends on *u* and on all input signals ${q}_{j}^{i}$. To emphasize this, the notation *v*[*u*; *c*_1_, *c*_2_, …, *c*_*k*_] will be used. If the classes are implicit, then a shorter form *v*[*u*] is more useful.

Every class of input signals *c*_1_, *c*_2_, …, *c*_*k*_ is represented by typical input signals ${c}_{i}=\{{q}_{1}^{i},{q}_{2}^{i},\cdots ,{q}_{{N}_{i}}^{i}\}$:

$$\begin{array}{rcl}{c}_{1} & = & \{{q}_{1}^{1},{q}_{2}^{1},\cdots ,{q}_{{N}_{1}}^{1}\}\\ {c}_{2} & = & \{{q}_{1}^{2},{q}_{2}^{2},\cdots ,{q}_{{N}_{2}}^{2}\}\\  & \vdots  & \\ {c}_{i} & = & \{{q}_{1}^{i},{q}_{2}^{i},\cdots ,{q}_{j}^{i},\cdots ,{q}_{{N}_{i}}^{i}\}\\  & \vdots  & \\ {c}_{k} & = & \{{q}_{1}^{k},{q}_{2}^{k},\cdots ,{q}_{{N}_{k}}^{k}\}\end{array}$$

If the input is a signal belonging to class *c*_*i*_, *e.g*. ${q}_{j}^{i}$ with *j* ∈ {1, 2, …, *N*_*i*_}, then the readout layer should report a corresponding class label *l*_*i*_.

A trajectory for a given combination of a drive signal *u* and an input signal ${q}_{j}^{i}$ can be written as

6 $$S[u,{q}_{j}^{i}](t)\equiv ({R}_{1}[u,{q}_{j}^{i}](t),{R}_{2}[u,{q}_{j}^{i}](t),\cdots ,{R}_{{N}_{R}}[u,{q}_{j}^{i}](t))$$

Such trajectories are traced during a finite time interval *t* ∈ [0, *T*] where *T* denotes the length of the observation time.

The separability index *v* is obtained by computing a typical distance between trajectories. A straightforward way of doing this it to compute the instantaneous distance between two trajectories $S[u,{q}_{j}^{i}](t)$ and $S[u,{q}_{j{\prime} }^{i{\prime} }](t)$ resulting from two inputs of different classes (*i* and *i*’) using the Euclidean norm in the state space:

7 $$||S[u,{q}_{j}^{i}](t)-S[u,{q}_{j}^{i}](t)|{|}^{2}=\mathop{\sum }\limits_{m=1}^{{N}_{R}}\,{({R}_{m}[u,{q}_{j}^{i}](t)-{R}_{m}[u,{q}_{j{\prime} }^{i{\prime} }](t))}^{2}$$

This distance will be denoted by

8 $${d}_{j,j{\prime} }^{i,i{\prime} }(t)=||S[u,{q}_{j}^{i}](t)-S[u,{q}_{j}^{i}](t)||$$

To obtain a typical distance one simply averages the instantaneous distance over time as

9 $${d}_{j,j{\prime} }^{i,i{\prime} }=\frac{1}{T}{\int }_{0}^{T}\,dt\,{d}_{j,j{\prime} }^{i,i{\prime} }(t)$$

**Figure 5:**
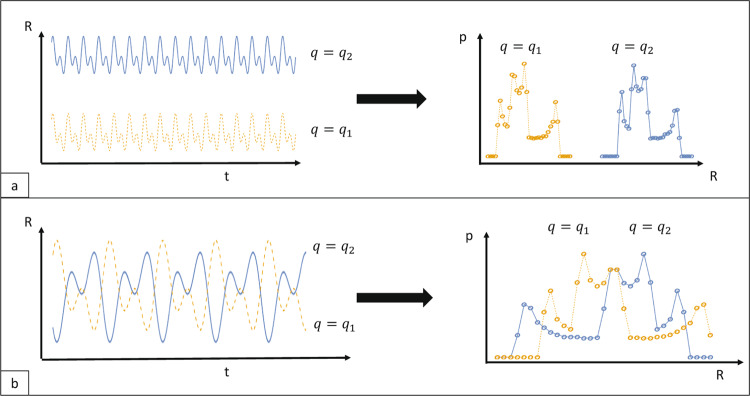
In the panels *a* and *b* two imaginary examples are used to show the probability distributions of the state variable *R* under two input signals *q*_1_ and *q*_2_. In the left side of the panels, the trajectories in the state space *R* are depicted. In the right side the resulting probability distributions of the state variable are shown.

The above formulas seem perfectly reasonable. However, there is a problem with these formulas that is not obvious. For example, consider the trajectories depicted in Fig. 5. Panel (a) shows a desirable behavior, a trademark of good input separation. By observing the probability distributions, one can with a bare eye decide a decision boundary. However, the behavior illustrated in panel (b) is problematic. For these trajectories the corresponding distance ${d}_{j,j{\prime} }^{i,i{\prime} }(t)$ is larger than zero for most time-points *t* implying that the average in Eq. (9) would be large too. This would signal that the behavior depicted in panel (b) is a trademark of good input separation and yet it would be hard to find a decision boundary by considering the probability distributions. The problem is that even though the individual trajectories are separated in time the regions they visit change over time. This implies that one would need to design a readout layer that is aware of these changes, leading to additional computational overhead.

A better distance estimate is obtained if one averages over time first, before the Euclidean norm is computed. The algorithm used to compute the typical distances is given by

10 $${d}_{j,j{\prime} }^{i,i{\prime} }=\frac{1}{\sqrt{{N}_{R}}}\Vert \bar{S}[u,{q}_{j}^{i}]-\bar{S}[u,{q}_{j{\prime} }^{i{\prime} }]\Vert $$

where

11 $$\bar{S}[u,{q}_{j}^{i}]\equiv ({\bar{R}}_{1}[u,{q}_{j}^{i}],{\bar{R}}_{2}[u,{q}_{j}^{i}],\cdots ,{\bar{R}}_{{N}_{R}}[u,{q}_{j}^{i}])$$

with

12 $${\bar{R}}_{m}[u,{q}_{j}^{i}]=\frac{1}{T}{\int }_{0}^{T}\,dt{R}_{m}[u,{q}_{j}^{i}](t)$$

The above equations result in the following compact expression

13 $${d}_{j,j{\prime} }^{i,i{\prime} }=\sqrt{\frac{1}{{N}_{R}}\mathop{\sum }\limits_{m=1}^{{N}_{R}}\,{({\bar{R}}_{m}[u,{q}_{j}^{i}]-{\bar{R}}_{m}[u,{q}_{j}^{i}])}^{2}}$$

Equation (13) is a more useful measure than the one given in Eq. (9). For example, consider the two trajectories in Fig. 5 panel (a). There the regions claimed by the trajectories are stable. The problem with Eq. (9) is that it cannot properly distinguish between the situations depicted in panels (a) and (b). This equation predicts large degree of separability for both panels, which is clearly incorrect. If Eq. (13) is used, then the distance measure is larger for the pair of trajectories in (a) than in (b).

Further, note that in Eq. (10) the additional factor, $1/\sqrt{{N}_{R}}$, has been added. This factor is extremely important. It penalizes careless increase of the dimension of the state space. In practical terms, without that factor, the distance between trajectories would increase when adding additional copies of the existing memristors but without any direct coupling with the existing network. Note that if such elements are added then they contribute to the sum in the distance formula and the distance measure increases. This wrongly signals an increase in the information processing ability of the device. In principle, such parallel copies of non-interacting elements should not add any additional information processing ability.

#### Combining typical distance between trajectories into an overall separability measure

Assuming that the typical distances have been estimated, there are several ways to define the index *v*. For example, the most natural definition would be to calculate the average over the typical distances:

14 $$\hat{\nu }[u;{c}_{1},{c}_{2},\cdots ,{c}_{k}]=\frac{1}{{N}_{D}}\mathop{\sum }\limits_{\begin{array}{c}i=1\end{array}}^{k}\,\mathop{\sum }\limits_{i{\prime} =i+1}^{k}\,\mathop{\sum }\limits_{j=1}^{{N}_{i}}\,\mathop{\sum }\limits_{j{\prime} =1}^{{N}_{i{\prime} }}\,{d}_{j,j{\prime} }^{i,i{\prime} }$$

where *N*_*D*_ denotes the number of all possible distances in the set of the training data. This number is given by

15 $${N}_{D}=\mathop{\sum }\limits_{\begin{array}{c}i=1\end{array}}^{k}\,\mathop{\sum }\limits_{i{\prime} =i+1}^{k}\,\mathop{\sum }\limits_{j=1}^{{N}_{i}}\,\mathop{\sum }\limits_{j{\prime} =1}^{{N}_{i{\prime} }}\,1$$

If each class contained *N* elements, then this number would be given by $${N}_{D}=(\begin{array}{c}k\\ 2\end{array}){N}^{2}$$. If the number of elements per class varies Eq. (15) is the only way to compute *N*_*D*_.

We argue that the procedure to estimate the separability index given in Eq. (14) is not the optimal one. Instead, in this study, the following estimate is used:

16 $$\nu [u;{c}_{1},{c}_{2},\cdots ,{c}_{k}]={(\mathop{\prod }\limits_{\begin{array}{c}i=1\end{array}}^{k}\mathop{\prod }\limits_{i{\prime} =i+1}^{k}\mathop{\prod }\limits_{j=1}^{{N}_{i}}\mathop{\prod }\limits_{j{\prime} =1}^{{N}_{i{\prime} }}{d}_{j,j{\prime} }^{i,i{\prime} })}^{\frac{1}{{N}_{D}}}$$

If the index *v* is calculated according to Eq. (14) then it can have a large value even though some distances are extremely small. This measure cannot be used if we prefer that all the distances are fairly large. However, if the Eq. (16) is used, the index *v* is maximized only if all distances are fairly large, since when one of the distances is very small, the final product will also be small.

### Genetic algorithm optimisation

Genetic algorithms (GA) are used in the first phase of the training procedure with the separability index *v* as a fitness function. GA is a strong optimisation technique that can be used to solve problems regardless of their complexity^35–37^. Additionally, there is one other reason why GA is preferred in this work and not other methods such as gradient-based optimisations: At the moment, calculating gradients of index *v* with respect to the parameters of the drive signal requires extensive mathematical analysis and validation since similar work has not been implemented before.

Genetic algorithm optimisation is implemented in which a combination of two nested loops is used. The small loop represents the typical GA optimisation steps (*e.g*. crossover or mutation). Iterations in this block are stopped when the best fitness does not improve in four consecutive steps. However, each GA block starts with random sampling of the candidate solutions. There is a possibility that the small loop does not converge to a solution with a sufficiently large fitness due to extremely unlucky choice of the candidates. To guard against that problem, we repeat the GA iteration block several times, remembering the best solution. If indeed the best fitness does not improve in two subsequent iterations then it is extremely likely that the best solution has been found. The ideas discussed above have been implemented as follows:

- 1. Determine the parameters to be optimised.
- 2. Big loop – **Repeat**:
  - 2.1. Initialization: Sample 300 candidate solutions and store in the list CANDIDATES. If a best candidate solution is available from the previous iteration (stored in the variable BEST) include it into the CANDIDATES list.
  - 2.2. Small loop – **Repeat**:

- 2.2.1. Keep the 30 best solutions out of the list CANDIDATES to use them for the genetic operations.
- 2.2.2. Choose another 10 randomly generated parents as candidate solutions.
- 2.2.3 Perform genetic operations with the 30 + 10 = 40 parents to produce 300 offsprings. Decide with probability 0.5 if you will perform mutation or crossover. Create an offspring in the following way.

- If you perform mutation, choose randomly 1 out of the 40 parents. Choose randomly one parameter of the parent. Convert it into a binary number and choose randomly one bit of the binary number to mutate. Then convert the mutated binary number into a decimal number.
- If you perform crossover, choose randomly 2 out of the 40 parents. Choose randomly one parameter in the same position for both parents. Convert both parameters into binary numbers. Crossover the binary numbers to produce a new binary number. Convert the new binary number into a decimal one.

- 2.2.4 Store the 300 offsprings and the 40 parents to the candidate solution list CANDIDATES. Note that this replaces all the elements and extends the size of the list by 40. After this step the length of the CANDIDATES is 340.
- 2.2.5 Sort the CANDIDATE list and pick the best candidate solution and store in the variable BEST.
  - 2.3. **Until** four subsequent iterations of the small loop give the same solution variable BEST.
  - 2.4. Continue with the big loop iteration.

- 3. **Until** two subsequent iterations of the big loop give the same best solution (the variable BEST does not change across two subsequent iterations).
  Classical GA suffers from premature convergence. This can happen if the population diversity is not maintained and the genetic operations of crossover and mutation cannot produce better off-springs than their parents^38^. In the GA scheme above, the following measures are taken to prevent premature convergence:
- In the step 2.1, a big amount of initial candidate solutions (300) are sampled.
- In the step 2.2.1, a big amount of best solutions (30) are kept to be used for genetic operation at the next iteration.
- In the steps of the small loop, a bigger priority is given to the solutions with best fitness and a slow convergence is avoided because 30 best solutions are chosen against 10 randomly generated. The randomly generated solutions encourage the genetic diversity which is important for avoiding premature convergence.

### Numerical simulations

This section provides details on how the simulations have been implemented. The drive signal is expressed as a Fourier series

17 $$u(t)=\frac{{A}_{0}}{2}+\mathop{\sum }\limits_{n=1}^{{n}_{c}}({A}_{n}\,\sin \,n\omega t+{B}_{n}\,\cos \,n\omega t)$$

when the drive signal is optimised, both the expansion parameters and the base frequency *ω* are optimised. With *n*_*c*_ = 4 there are 10 parameters in total. The fixed numerical values of the Pershin-Di-Ventra model parameters are shown in Table 4. When the input layers were not optimised, the parameters *m*_1_, *m*_0_, *k*_1_ and *k*_0_ were fixed. Their fixed values are shown in the Table 5.

**Table 4:** Pershin Di-Ventra model.

	numerical value
*β*_*c*_	3.0
*α*	1.0
*V*_*thr*_	0.5
*R*_*min*_	1.0
*R*_*in*_	4.0
*R*_*max*_	7.0

**Table 5:** Numerical values of the parameters *m*_1_, *m*_0_, *k*_1_ and *k*_0_.

	numerical value
*m*_1_	0.83
*m*_0_	5.5
*k*_1_	3.33
*k*_0_	5.0
